# A modified two-incision technique for primary total hip arthroplasty

**DOI:** 10.4103/0019-5413.41850

**Published:** 2008

**Authors:** B Sonny Bal, Santaram Vallurupalli

**Affiliations:** Department of Orthopedic Surgery, University of Missouri, Columbia, USA

**Keywords:** Minimally invasive surgery, total hip arthroplasty, two-incision surgery for THA

## Abstract

**Background:**

Minimally invasive surgery can be technically demanding but minimizes surgical trauma, pain and recovery. Two-incision minimally invasive surgery allows only intermittent visualization and may require fluoroscopy for implant positioning. We describe a modified technique for primary total hip arthroplasty, using two small incisions with a stepwise approach and adequate visualization to reliably and reproducibly perform the surgery without fluoroscopy.

**Materials and Methods:**

One hundred and two patients with an average age of 60 years underwent modified two-incision minimally invasive technique for primary THA without fluoroscopy. The M/L taper femoral stem (Zimmer, Warsaw, IN) and Trilogy (Zimmer) hemispherical titanium shell, with a highly cross-linked polyethylene liner, was used. Operative time, blood loss, postoperative hospital stay, radiographic outcomes and complications were recorded.

**Results:**

The mean operating time was 77 min. The mean blood loss was 335 cc. The mean hospital stay was 2.4 days. Mean cup abduction angle was 43.8°. Mean leg length discrepancy was 1.7 mm. Thirteen patients had lateral thigh numbness and two patients had wound complications that resolved without any treatment.

**Conclusion:**

A modified two-incision technique without fluoroscopy for primary total hip arthroplasty has the advantage of preserving muscles and tendons, shorter recovery and return to function with minimal complications. Provided that the surgeon has received appropriate training, primary total hip arthroplasty can be performed safely with the modified two-incision technique.

## INTRODUCTION

The attractiveness of less surgical trauma, earlier return to function and patient demand will continue to drive innovation in minimally invasive total hip arthroplasty surgery.^1^ Minimally invasive total hip arthroplasty with two small incisions has been associated with excellent short-term outcomes^2^,^3^ and improved patient recovery with some patients leaving the hospital on the day of surgery. Others have encountered a high incidence of complications like femur fracture, component malpositioning, nerve palsy and repeat surgery.^4^–^6^ There is agreement among investigators that minimally invasive hip arthroplasty is technically demanding and is associated with a steep learning curve that can lead to increased complications, at least early in the surgeon's experience.^7^,^8^ Therefore, the challenge is to identify and develop safe surgical techniques that deliver the benefits of minimally invasive surgery while ensuring safety, reliability and reproducibility.

In this report, we describe a modified version of the fluoroscopically-assisted two-incision technique originally described by Mears and Berger^5^ to perform primary total hip arthroplastys. The surgical approach is unchanged from that described by previous authors; instead, our modifications to the procedure itself permit surgery without fluoroscopy and stem insertion without femoral canal reaming. The short-term outcomes in a consecutive group of patients are presented, along with potential pitfalls and strategies to simplify the operation.

## MATERIALS AND METHODS

One hundred and two patients (102 hips) underwent primary total hip arthroplasty (THA) using the technique described below. All having failed reasonable conservative therapy and presenting with radiographic evidence of severe hip arthritis and were candidates for primary THA were included in the study. The diagnosis of hip disease was osteoarthritis in 89 patients, osteonecrosis in seven and rheumatoid arthritis in three. In the remaining three patients, the etiology of hip arthritis was uncertain. No patient was excluded on the basis of weight; the mean patient BMI in this series was 31.1 (18.3-60.8). Only two patients who were otherwise candidates for primary THA were excluded from this series; both had acetabular dysplasia that possibly required bone grafting using a surgical approach different from the two-incision technique.

### Operative procedure

#### Templating

**Figure 1:**
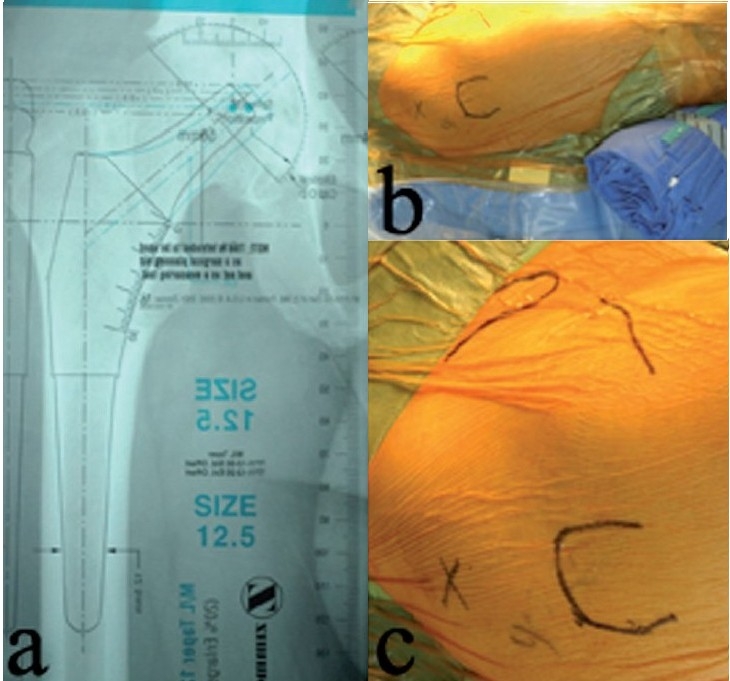
Preoperative templating (a) allows the surgeon to predict the implant sizes, prosthetic neck length and whether or not an offset femoral stem will be necessary. A sterile roll (b) is placed under the knee to flex the hip to about 30-45° throughout the procedure. The medial border of the tensor fascia lata muscle (c) is palpated and the anterior incision is marked along this border, along a line connecting the tip of the trochanter to the ASIS

Preoperative templating is a critical component because of limited surgical exposure with the minimally invasive two-incision technique [Figure 1a]. Implant sizes, prosthetic neck length and extent of femoral offset can be predicted by templating.^9^ Leg lengths following arthroplasty can also be determined by defining the level of the calcar cut relative to the lesser trochanter on the AP hip radiograph.

#### Patient positioning and draping

Following induction of spinal anesthesia, the patient is placed supine on a standard operating table with a radiolucent extension. A radiolucent extension on the table is helpful if radiographic imaging is needed, although the present technique does not require such imaging. The operative hip is positioned as close as possible to the edge of the table, with a folded towel placed under the midsacrum to elevate the pelvis slightly. The ipsilateral arm is padded and taped securely over the anterior chest. The patient positioning is similar to that for a supine femoral nailing.^10^ Two self-adhesive EKG electrodes are placed on the medial malleoli of the ankles; palpation of these allows assessment of leg lengths.

Skin preparation should be from the ipsilateral knee to one hand-breadth above the iliac crest. The leg is draped circumferentially from above the iliac crest to the knee joint. After draping, a sterile roll is placed under the knee to flex the hip [Figure 1b]. Hip flexion relieves tension on the femoral nerve and eases anterior retraction, thereby facilitating acetabular exposure in this technique. The greater trochanter and the ipsilateral anterior superior iliac spine (ASIS) are palpated and their positions are marked with a skin marker. Anteriorly, the medial border of the tensor fascia lata muscle is palpated and the anterior incision is planned along this border [Figure 1c]. The ASIS and electrodes previously placed on the ankle malleoli are palpated through drapes to verify pelvic position and existing leg lengths.

#### Anterior dissection

Surgical landmarks are easily palpable; these consist of the greater trochanter and the anterior superior iliac spine; both bony prominences can be felt by palpation. Along the lateral thigh, the femoral shaft can be similarly palpated. Anteriorly, the medial border of the tensor fascia lata can be similarly identified. Further medially, the pulsations of the femoral artery are palpable and this position should be marked on the surgical field. A 5-7 cm anterior incision is placed directly over the femoral neck with the hip flexed at 30 to 45°. Until the surgeon has gained enough confidence to judge proper incision location, fluoroscopy can be used to localize the anterior incision; this should be over the femoral neck. Skin flaps are developed and elevated with retractors. The subcutaneous tissues are spread with scissors to preserve branches of the lateral femoral cutaneous nerve, which are usually visible in the surgical field. Nerve branches are isolated medially and the dissection is carried on their lateral side. This lateral incision and blunt dissection avoids the major part of the lateral femoral cutaneous nerve, which is located superficial to the sartorius.

**Figure 2:**
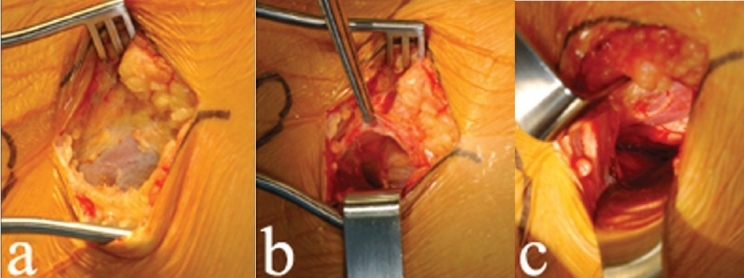
The fascia overlying the tensor muscle (a) is incised to expose the muscle belly of the tensor. The cobra retractor (b) is placed between the tensor and the hip capsule. A 90° retractor (c) is used to pull the skin and soft tissues medially

The fascia overlying the tensor muscle is less developed than the fascia lata on the lateral thigh [Figure 2a]. This fascia is translucent and thin; it is incised to expose the tensor muscle belly. Verify that the muscle so exposed is the tensor and not the sartorius, because in some muscular individuals, both these muscles may be well developed, leading to possible confusion. Each of these muscles in enclosed in its own fascial sheath and the desired dissection is in the internervous plane between the sartorius and the tensor, as originally described by Smith-Petersen.^11^

If the tensor muscle has been correctly identified, the medial border is easily isolated with the palpating finger; the superior (cephalad) hip joint capsule lies just deep to this location. An advantage of this technique is that the hip capsule can be accessed easily, even in the obese patient, since it lies close to the anterior thigh and is easily exposed by spreading the overlying muscles. A cobra retractor is placed between the tensor and the hip capsule [Figure 2b]. A 90-° retractor is used to pull the skin, soft tissues and sartorius medially [Figure 2c]. In this interval, blunt dissection distally will expose the ascending branches of the lateral femoral circumflex artery and vein, which must be coagulated to avoid later bleeding. With a palpating finger, the plane between the hip capsule and the abductors is now clearly defined. Using a sharp elevator, the muscle origins off the hip capsule are dissected medially and a lighted Hohman retractor is placed around the inferior capsule.

A second Hohman retractor is placed on the bony rim of the anterior acetabulum. Once the entire anterior hip capsule is cleared and visible, it can be cut and excised with cautery or a scalpel. Excision of the anterior capsule improves exposure and the hip capsule does not appear to contribute to anterior hip stability.^12^ In order to prevent bleeding, the entire anterior hip capsule should be cleared of soft tissues, muscle attachments and visualized cleanly before it is excised.

**Figure 3:**
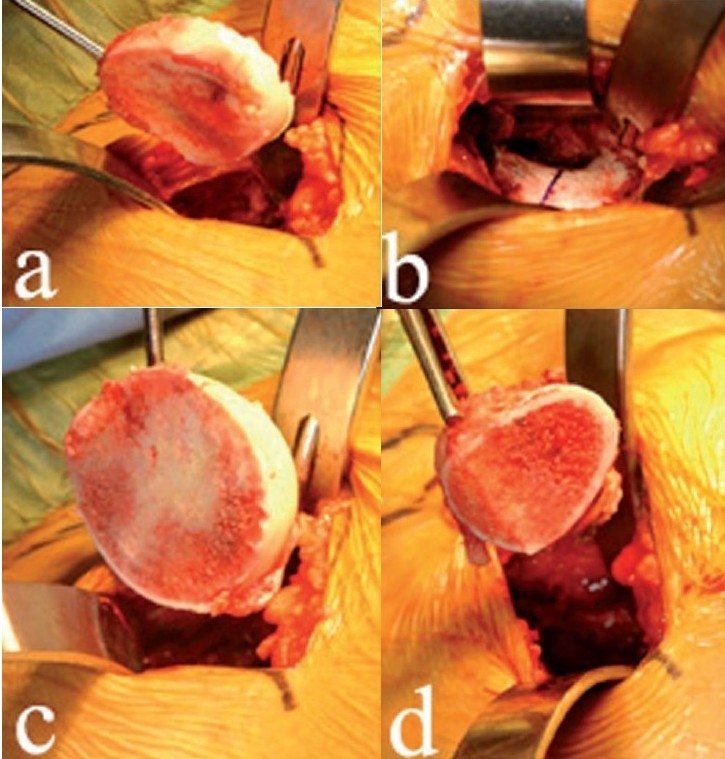
A threaded Steinmann pin (a) is used to capture the osteotomized femoral neck segment. Slight distraction of the (b) leg assists in removing the head from the acetabulum. In order to approximate the position of the planned cut on the preoperative templates, a pen mark (c) is made proximal to the lesser trochanter The calcar fragment (d) is removed without cutting through the trochanter

Prominent osteophytes on the anterior-superior rim of the acetabulum should be removed with an osteotome. The leg is rotated to visualize the position of the femoral head and neck and two cuts are made in the femoral neck, just distal to the femoral head, about 10 mm apart. To make the femoral neck cuts safely, an oscillating saw is used to notch the anterior cortex of the femoral neck, followed by a reciprocating cut to divide the bone. A threaded Steinmann pin is used to capture and remove the neck fragment. Removal is easy with slight distraction of the leg and mobilization of the neck fragment [Figure 3a].

The femoral head fragment is next captured with a threaded Steinmann pin and a curved instrument (Zimmer, Warsaw, IN) designed to divide the ligamentum teres, is passed around the head. As with the neck fragment, slight distraction of the leg assists in mobilizing the head and removing it from the acetabulum [Figure 3b].

Next, the hip is flexed and adducted by placing the leg in a figure-of-four position. With external rotation, the lesser trochanter is palpated and a lighted Hohman retractor is placed around it. A cobra retractor is placed lateral to the trochanteric ridge. The residual anterior hip capsule is removed from the femur and dissection with the cautery knife is carried medially, until the lesser trochanter is visualized. This is a key step, since the lesser trochanter is a bony landmark previously identified during templating. By making the calcar cut relative to this landmark, previously templated leg length relationships can be reproduced. A skin marker can be used to illustrate the placement of the calcar cut [Figure 3c].

The final calcar cut is made from medial to lateral, under direct visualization and stopping close to the greater trochanter. A second, vertical cut is made under direct visualization to meet the first cut and the calcar fragment is removed, without cutting through the greater trochanter [Figure 3d]. Inadvertent damage to the greater trochanter can occur if the two cuts are performed blindly. If the hip has an external rotation contracture, the leg can be rotated externally and the posterior hip capsule is released off the proximal femur with the cautery tip.

#### Acetabular preparation

**Figure 4:**
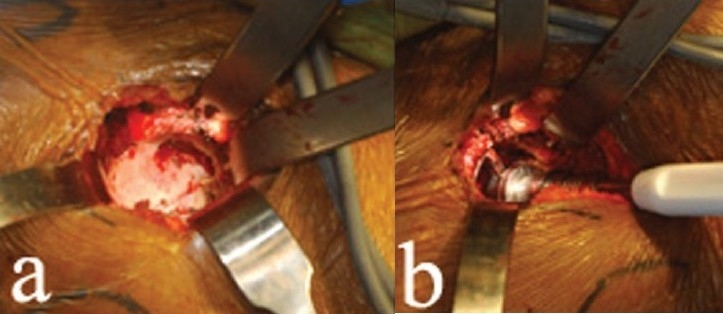
With the femoral neck now removed, retractors (a) can be adjusted to gain excellent visualization of the entire acetabulum. Surgeons must be careful to avoid levering the reamer arm on the soft tissues of the thigh and thereby reaming out the anterior wall of the acetabulum (b)

With the femoral neck and head excised, a cobra retractor is placed in a superior-posterior position on the acetabular rim and a lighted Hohman is placed on the anterior rim. A second Hohman is placed further inferiorly and the retractors can be adjusted to obtain visualization of the entire acetabulum [Figure 4a]. The acetabular labrum is removed and the bony socket is cleared of any soft tissue and osteophytes.

Reaming is done with hemispherical reamers modified and optimized for minimally invasive hip arthroplasty. The position of the acetabular notch can orient reaming, especially if the surgeon is unfamiliar with the supine position. Care must be taken to avoid levering the reamer arm on the anterior thigh and thereby reaming out the anterior socket [Figure 4b]. The acetabular exposure is at least as good as that with any other surgical approach used in THA. If needed, a slight distraction to the leg will further improve acetabular visualization and reaming.

**Figure 5:**
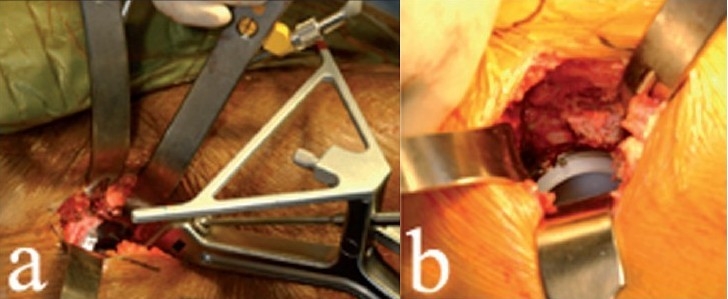
External alignment guides (a) fit on the inserter, helping to ensure proper cup orientation. A slight distraction on the femur (b) facilitates polyethylene liner insertion

An uncemented acetabular component is inserted, although cemented cups can be used as well. An offset cup inserter facilitates implantation, although standard inserters can also be used with this approach. Cup orientation is determined by external alignment guides that fit on the inserter and by direct visualization [Figure 5a]. If the surgeon is unfamiliar with the supine position, the usual error is to place the cup in excessive anteversion. Fluoroscopy can be misleading in determining cup position, unless the surgeon is aware of the amount of pelvic tilt and rotation and can compensate for such in interpreting fluoroscopic images. The polyethylene liner insertion is done as for standard techniques, although slight distraction on the leg will facilitate liner implantation [Figure 5b]. If polyethylene liner insertion is difficult, it is usually because of retained osteophytes at the periphery of the bony socket.

#### Femoral preparation

**Figure 6:**
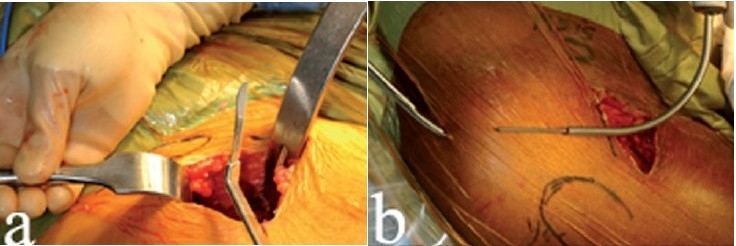
A bent #10 scalpel blade (a) is used to make a capsulotomy about 2-3 cm in length, from the superior acetabular rim to the tip of the greater trochanter. Where the guide tents the posterior skin and in line with the position of the femur, a stab incision is made (b) to introduce long, curved scissors through the skin

In the present technique, femoral stem implantation is similar to the insertion of a femoral nail for a femur fracture. The superior hip capsule, inserting at the tip of the greater trochanter is palpated and visualized by retracting the tensor with a 90° retractor. Distraction on the leg stretches the capsule and improves visualization. A bent #10 scalpel blade is used to make a capsulotomy about 2-3 cm in length, from the superior acetabular rim to the tip of the greater trochanter [Figure 6a]. This gap in the capsule allows passage of the femoral instruments and implant. Through this capsular gap, the palpating finger can identify an interval just posterior to the abductors, through which a U-shaped guide is passed, with the leg in a figure-of-four position.

Where the U-shaped guide tents the posterior skin and in line with the axis of the femur, a second incision is created and through this long, curved scissors are passed [Figure 6b]. The closed scissors' tip is now passed into the hip joint, posterior to the abductors and aimed at the femoral canal. Visualization of the hip joint space through the anterior incision ensures the safety of this step. As the scissors are withdrawn from the hip joint, the soft tissues are spread gently by opening the scissors to create a pathway for the femoral broaches. Further dissection and definition of this passage can be done with blunt finger dissection. The posterior skin incision is now extended to about 1 inch in length and if these steps are properly performed, a palpating finger should readily pass from the posterior incision into the hip cavity.

**Figure 7:**
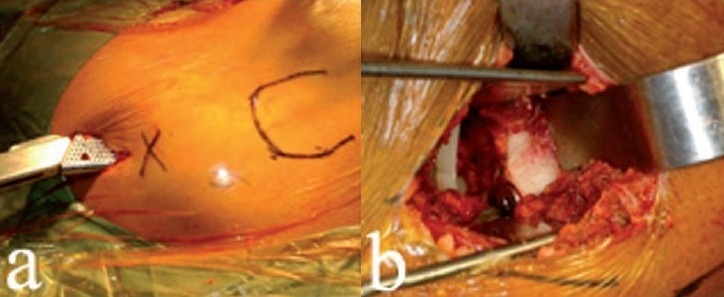
Broaching is started by pushing the smallest broach into the femoral canal (a). The broach handles are specially designed and optimized for this approach. During broaching, two lighted Hohmans (b) are placed around the calcar, providing excellent visualization of the femur

A canal reamer is used to open the femoral canal and broaching is started by pushing the smallest broach into the femoral canal [Figure 7a]. The broach handles are specially designed and optimized for this approach. Broach anteversion is determined in three ways. First, a Steinmann pin is placed in the specially designed broach handle and the pin is aligned with the patella. Second, the calcar is visualized directly as the broach is introduced into the femoral canal. Finally, if the smallest broach is firmly pushed into the proximal femur, it will seat itself in the proper anteversion. During broaching, two lighted Hohman retractors are placed around the calcar, to improve femur visualization [Figure 7b]. Femoral canal reaming is unnecessary with this type of taper stem.

We have used the M/L taper femoral stem (Zimmer, Warsaw, IN), which is a common design, consisting of a porous-coated, collarless, wedge-shaped implant that is placed in the femur by broaching the canal, without reaming. If a broach will not advance, use the broach as a rasp to cut the endosteal bone. With patience, the broach will advance, without forceful impaction. With progressive rasping, the broach can be advanced till it is flush with the calcar. While broaching, a lateral pressure must be maintained so that the femoral implant is properly lateralized. These steps are similar to those used with standard total hip arthroplasty; we have found intraoperative fluoroscopy to be misleading in femoral stem placement.

With the final broach removed, the hip cavity is irrigated to clear out any debris, in preparation for femoral component implantation. A bone hook placed around the proximal femur is used to distract the femur laterally, as an assistant applies traction to the leg. Visualizing the relationship between the acetabular component and the cut femur gives the surgeon an indication of the offset and length of the anticipated reconstruction and confirms preoperative templating. The femoral implant is now impacted into the canal under direction visualization. The posterior skin incision must usually be stretched over the stem taper and any obstructing fascial bands can be divided bluntly to advance the stem through the soft tissue passage into the hip cavity. Final stem seating should be close to that of the last broach used, relative to the calcar cut. If a fracture of the calcar develops during stem placement, the stem should be withdrawn slightly and a 2-mm drill hole should be made in the lesser trochanter by externally rotating the femur. In this hole, a cerclage cable or wire is passed around the femur and tightened. Fracture stabilization with this method is not difficult if the lesser trochanter has been exposed in the previous steps. Furthermore, if the calcar and lesser trochanter are in direct vision during broaching and stem insertion, the risk of an unrecognized proximal femur fracture is less.

**Figure 8:**
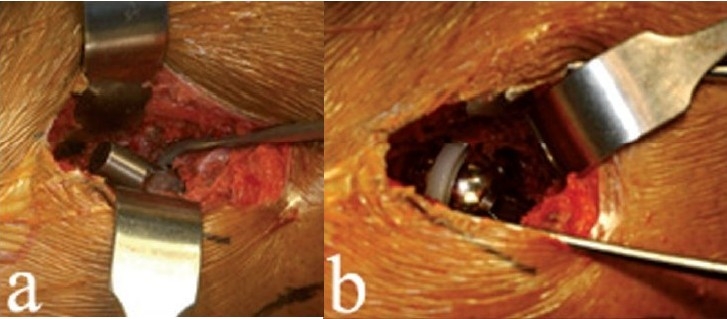
External rotation of the femur (a) facilitates exposure of the femoral component taper. (b) Once the trial femoral head is on the taper, leg distraction and control of the femur with the bone hook facilitates hip reduction

Once the stem is seated, the taper is cleaned and trial femoral heads are used. We begin with the shortest neck first and use a 36-mm diameter femoral head whenever possible. Exposure of the femoral component taper is facilitated by external rotation of the femur and by lifting the femur up with the bone hook [Figure 8a]. Once the trial femoral head is on the taper, leg distraction and control of the femur with the bone hook facilitates hip reduction [Figure 8b].

Hip stability and leg lengths are next assessed and the proper length of femoral neck is chosen. Leg lengths are determined by palpating the previously placed markers on the medial malleoli. If the calcar cut is made as templated with respect to the lesser trochanter and if the last femoral broach used is seated flush with the calcar cut, the final prosthetic neck size is invariably within one size of the templated measurement.

#### Wound closure

**Figure 9:**
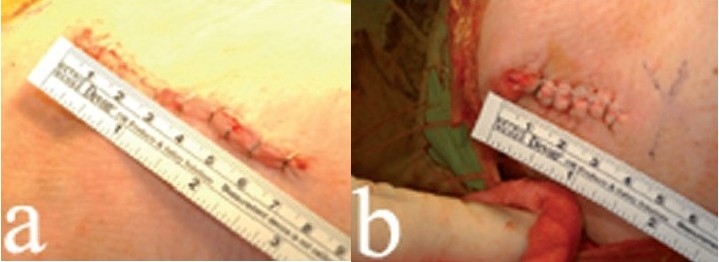
The typical anterior incision length (a) and typical posterior incision (b)

The anterior incision is closed over a drain. When closing the fascia over the tensor muscle, only the minimum number of sutures should be used, with care taken to capture only the fascial edges in the stitches. This will avoid incarceration of small branches of the lateral femoral cutaneous nerve. The posterior incision usually needs only a single stitch to close the subcutaneous layer, followed by staples for both incisions. We inject 0.25% bupivacaine along the skin incisions. The typical anterior incision length [Figure 9a] and the posterior incision [Figure 9b] is illustrated.

#### Postoperative care and rehabilitation

For prophylaxis against deep venous thrombosis in uncomplicated patients, we use a multimodal regimen consisting of intravenous heparin given once when the femoral canal is broached, followed by oral aspirin given twice daily for eight weeks, in combination with early patient mobilization, ankle exercises and intermittent foot pumps.

Narcotic and anti-inflammatory medications are started before surgery and administered regularly for the first 48 h after surgery. The drain is removed on the first or second day after surgery. Physical therapy and patient mobilization are initiated on the first postoperative day. No specific protocol is used to encourage early discharge from the hospital and each patient's response to the surgery determines discharge planning.

All patients are instructed to use a walker or two crutches and advance weight-bearing and exercises as tolerated until one month after surgery. Thereafter, patients progress to a cane if needed. Aside from these precautions, no other restriction is placed on the patient, nor is physical therapy routinely prescribed.

## RESULTS

One hundred and two consecutive patients (102 hips) underwent primary hip replacement using modified two-incision technique without fluoroscopy. Mean patient age was 60.6 years (37-88 years); there were 61 males and 41 females with a mean body mass index of 31.1 (18.3-60.8). The right side was affected in 56 cases while left in 46. Clinical and radiographic outcomes were evaluated specifically to identify any acute complications in this group at a mean follow-up of 10 months after surgery (range 8-14 months).

Mean operative time was 77 min (range 66-127 min), measured from the placement of the first skin incision to completion of skin closure. None of the skin incisions had to be extended during surgery and the surgical approach did not have to be altered in any case. Mean hospital stay was 2.4 days (range 2-6 days); no patient required in-patient rehabilitation or referral to a physical therapist following discharge. In terms of complications, 13 patients (12.7%) developed numbness on the lateral thigh and in all 13 cases, symptoms had to be elicited by questioning and examining the patient. None of the patients offered complaints of thigh numbness, which either disappeared or was present only minimally at the most recent follow-up. In two patients (2%), the proximal end of the anterior incision had serous drainage that persisted for several days after discharge from the hospital; neither one required antibiotics nor repeat surgery. No component required re-positioning after implantation and no intraoperative fractures were encountered in the present series.

On follow-up radiographs, none of the components had migrated from the position noted on comparable images taken after surgery. Mean acetabular cup abduction was 43.8° (39.7-48.5°), with no component outside the ideal range of 30-50° of abduction.^13^ All acetabular components were seated in the bony socket, with no radiolucent lines were present at the dome of the metal cup on the postoperative radiographs. Only 2 patients developed incomplete radiolucent line between cup and pelvis. All femoral components were within 3° of varus or valgus alignment relative to the diaphyseal femoral canal. Leg lengths were compared during surgery and existing length relationship was restored. Mean leg-length discrepancy was 1.7 mm (range −1.3 mm to + 3.7 mm).

## DISCUSSION

The operation described here was modified from that described by proponents of the two-incision technique for THA.^2^,^14^ One modification was that fluoroscopy was not used to guide component placement.^15^ Instead, we relied on direct visualization of key landmarks, in combination with external guides to properly position implants, just as in standard hip replacements. In our experience, intraoperative fluoroscopy can be misleading and unreliable in positioning the implants correctly. Direct visualization of the acetabulum and proximal femur and the use of external alignment guides helped us avoid two complications that have been associated with the two-incision technique, i.e., component malpositioning and proximal femur fractures.

Another modification was that we implanted an M/L taper femoral stem that did not require reaming of the femoral canal, which is associated with less blood loss. This stem is easier to implant through a smaller incision and has excellent long-term outcomes.^16^

If the surgeon is unfamiliar with THA in the supine position and with the use of minimally invasive surgical techniques, the procedure described should be undertaken with caution. Training at seminars, a thorough review of the relevant anatomy, knowledge of the complications associated with the procedure and the strategies to avoid them, cadaver training and observation of an experienced surgeon are necessary before performing two-incision THA. At least early on, a surgeon should plan on making longer incisions to ensure adequate surgical exposure until familiarity with the anatomy of the anterior thigh is gained. Further, since the procedure is easiest in female patients who are of average weight and size and without deformity of the hip joint, patient selection will facilitate early learning.

Anterior thigh numbness is a known outcome of any incision placed in the anterior thigh^11^,^17^,^18^ and it occurred at the rate of 13% in the present series. Thigh numbness resolved over time in each case. To avoid thigh numbness, blunt dissection is advisable once the anterior incision is placed and the subcutaneous tissues should be mobilized towards the medial side. This avoids division of the branches of the lateral femoral cutaneous nerve and also decreases traction on these branches during retraction.

The modified two-incision method described here is similar to the surgical approaches described by others^3^ except that we did not make separate skin openings to insert the acetabular reamers. We also relied on direct visualization and palpation of anatomic landmarks, such as the lesser trochanter, acetabulum and calcar, to orient the surgeon and to guide the prosthesis implantation. Proper patient positioning and careful preoperative templating, while important in any THA, were critical to the safety and success of the modified two-incision procedure. The procedure described here is a stepwise exercise in surgical planning and execution, with each step relying on successful completion of the prior steps. The duration of surgery is slightly increased over standard methods, but not significantly so and the outcomes in terms of reduced pain and earlier return to function and activity are remarkable advantages of this technique.^2^,^3^,^6^,^14^,^15^
